# Myeloperoxidase, a possible biomarker for the early diagnosis of cardiac diastolic dysfunction with preserved ejection fraction

**DOI:** 10.1080/14756366.2018.1499626

**Published:** 2018-08-31

**Authors:** Bogdan Ioan Coculescu, Gabi Valeriu Dincă, Constantin Bălăeţ, Gheorghe Manole, Maria Bălăeţ, Cristina Mariana Stocheci

**Affiliations:** a Faculty of Medicine, Titu Maiorescu University, Bucharest, Romania;; b Center for Military Medical Scientific Research, Bucharest, Romania;; c Faculty of General Nursing, Bioterra University, Bucharest, Romania;; d Lil Med Clinic, Bucharest, Romania;; e Clinical Hospital Colentina, Bucharest, Romania;; f Imperial College London, London, UK;; g Faculty of Sciences, University of Pitesti, Pitesti, Romania

**Keywords:** Myeloperoxidase (MPO), left ventricle ejection fraction (LVEF), preserved systolic function (PRESYF), diastolic dysfunction (DD), reactive oxygen species (ROS)

## Abstract

The current study was conducted on a sample of 91 patients diagnosed with diastolic dysfunction (DD) with preserved systolic function caused by a painful chronic ischaemic cardiopathy – angina pectoris stable at the effort. The diagnosis was established following anamnesis, electrocardiogram, and echocardiography. Myeloperoxidase (MPO) serum levels were assessed in all patients and then these values were correlated with some of the echocardiography parameters that proved the mentioned diagnosis.

In conclusion, the execution of this investigation triad (electrocardiogram, echocardiography, and MPO) allows:

- Stratifying the patients depending on the disease risk by early detecting of any possible DD with preserved systolic function.
- The use of the MPO increased circulating levels as a biomarker for diagnosis and risk due to the statistically significant correlation between those and the results of the other two aforementioned paraclinical investigation.

## Introduction

The myocardial contractile function is affected at a relatively early point during the evolution of cardiac diseases, on account of which the latest European medical regulations for heart failure considers as necessary, even in the subclinical stage, to assess the efficiency of not only the systolic but also the diastolic function^1^ ^,^ ^2^.

Regardless of whether the systolic debit is preserved or not, early detection of risk factors is useful for effective prevention strategies in avoiding premature cardiac insufficiency (CI) and in providing adequate treatment, including aetiological therapy. Clinical studies on CI reveal that myocardial performance depends on the left ventricle (LV) diastolic function, which is primordially dependent on the relaxation ability of the heart muscle^3^ ^,^ ^4^. In regard to this last process, it is known that the lusitropic status is determined by both biochemical and biomechanical (active relaxation) influences, as well as by biophysical properties of the heart (passive rigidity)^4–6^.

Since 1991, the medical practice has recognised a new clinical form of heart failure, diastolic dysfunction (DD)^7^. DD is defined as a clinical syndrome connected pathogenically to anomalous ventricular filling and relaxation, especially affecting the LV, which manifests initially with preserved systolic function (PRESYF). The defining characteristic of the disease is the preservation of the systolic debit (>45% than normal), whereas there are alterations in the diastolic function^8^.

According to the incidence rates, the main pathogenic mechanism responsible for the onset of CI with normal LV ejection fraction is myocardial ischaemia that slows down the heart muscle relaxation, reduces the diastolic distension, and remodels the heart concentrically increasing the width/radius ratio. Under hypoxia/ischaemia conditions, the LV distension problems arise as an expression of healthy arias and ischaemic, even fibrous lesions coexisting in the heart muscle, which alter the relaxation process and generate a delay and inhomogeneity of relaxation – increasing the phenomena, especially during effort^9^ ^,^ ^10^.

Cellular oxidative stress is defined as the biological state characterised by an excess concentration of oxidative agents (reactive oxygen species (ROS) and reactive nitrogen species (RNS)), which results as a consequence of either intensified synthesis or decreased antioxidant systems activity^9^ ^,^ ^11–13^.

In medical practice the pathogenesis induced by oxidative stress on the organic level is functionally evaluated to quantify the participation share, without regard of its primary source:

- Local production of oxidant agents by implicating the cell structures of the particular organ or/and the cells migrated or infiltrated at that level.
- Systemic synthesis in other tissues and organs^10^ ^,^ ^13^ ^,^ ^14^.

In the case of ischaemic cardiomyopathy induced by coronary atherosclerosis in the first category, that of the local sources producing oxidant agents, there are the myocardial fibres, myocytes, endothelial cells from the regional vessels, whereas in the other class, the one of the migrated cells or the figurate elements implicated in inflammatory processes are placed neutrophils and especially monocytes turned into macrophages^9–11^ ^,^ ^15^ ^,^ ^16^. The monocyte activation process includes the following three steps:

- Mobilisation of resident/responsive monocytes, from the central axis of laminar blood, flow into passing in the interstitial space by diapedesis.
- Initiating the stimulating process on the monocytes, which results in their partial activation.
- Complete activation of monocytes, which allows them to metamorphose into macrophages^9^ ^,^ ^11^ ^,^ ^13^ ^,^ ^16^.

Through the production of chemoattractant factors, the activated monocytes have implications in the myocardial oxidative stress evolution, ensuring the persistence and expand of inflammation that generates other chemoattractant factors on its own. The structural–functional destructive processes in the ischaemic heart muscle are mediated by at least 24 such factors among which are tumour necrosis factor (TNF), interleukin-1 (IL-1), and stimulating factors such as granulocyte-monocyte colony-stimulating factor (GM-CSF), granulocyte colony-stimulating factor (G-CSF), and monocyte colony-stimulating factor (M-CSF)^9^ ^,^ ^11^ ^,^ ^13^.

Although the consulted medical literature recognises unanimously the myeloperoxidase (MPO) serum levels as a biomarker for oxidative stress and endothelial dysfunction in CI, there are limited studies available on the possible significance of MPO as an indicator of inflammation and heart muscle remodelling processes^15^. Histochemically, the myocytes that are subject to ischaemia undergo lysis due to both autophagy (catalysed by the enzymes released by the myocardial fibre itself) and necrosis, produced by the mononuclear phagocytic system cells^11^ ^,^ ^13^ ^,^ ^16^. Although the last one is triggered as a local reconstructive process, it represents a destructive mechanism with implications in further inflammation development and fibrotic matrix proliferation. As MPO is a lysosomal monocyte-macrophage enzyme, it catalyses the reaction between hydrogen peroxide and chloride ion, leading to hypochlorite (ClO^–^), which turns into hypochlorous acid (HClO) and gains lytic properties over the cells^10^ ^,^ ^14^.

## Materials and methods

On the basis of the overall acceptance of serum MPO among the markers used in endothelial dysfunction management, the present study is aiming to investigate the possible signification of serum MPO as a biomarker for CI with DD and preserved ejection fraction (Table 1).

**Table 1.** Cardiac insufficiency (CI) biomarkers classification after the pathophysiological mechanism they are involved in^15^.

Category/axis of clinical importance	Enumeration of the approved biomarkers used in CI management
Myocytic stress (cardiac fibre stretch)	BNP, NT-proBNP, proadrenomeduline, sST2
Neurohormonal activation	Norepinephrine, renin, angiotensin II, aldosterone, vasopressin
Myocytic injury	Troponin I and T, CK-MB
Inflammation	PCR, TNF, APO-1, Interleukin 1, 6, and 18
Oxidative stress and vascular remodelling	Oxidised LDL, MPO, plasma and urinary isoprostanes, plasmatic malondialdehyde, serum uric acid
Extracellular matrix remodelling	Metalloproteinases, metalloproteinase tissue inhibitors, collagen propeptides
Renal injury	Creatinine, cystatin

The research sample consisted of 91 patients with a positive diagnosis of DD with preserved ejection fraction (PRESYF), having a unique aetiology caused by the only one aetiological factor, namely chronic ischaemic painful cardiopathy (CICD).

The preservation of the systolic debit was an important condition imposed on the participants in the study to limit the sources of oxidant agents production, only to those on the myocardial level, in the case of mal-irrigation. Because of the fact that it is demonstrated and universally acknowledged that in chronic CI with reduction of the systolic flow there occurs a decrease in the organ debit, in these conditions all structures of the organism are forced to function under state of hypoxia. From pathogenic point of view, the consequence is the appearance of new sources of ROS and NOS production – a possibility that has been avoided through the selection of patients with ischaemic cardiopathy, but conserved systolic debit^13^.

For each one of the 91 patients (35 women and 56 men) in the study sample, the aetiological diagnosis of the myocardial contraction deficiency (ischaemic cardiopathy) is justified by the presence of stable angina pectoris at effort, reported during anamnesis, and the paraclinical exploration results. To evaluate the functional condition of the ventricular myocardium during diastole all participants underwent:

- Resting state electrocardiogram and supplementary effort test if the first could not demonstrate any ischaemic modifications.
- Echocardiography – bi-dimensional evaluation, M-mode, pulsed Doppler (PW), and tissue pulsed Doppler (TDI). Referring to the clinical utility of echocardiography, the specialized literature considers this to be the best non-invasive method for assessing DD and LV filling pressure. The M-mode used in the current study was limited because the DD is suggested indirectly. In comparison, the other echocardiographic techniques such as the transmitral blood flow Doppler and the tissue Doppler are more sensitive and more specific, which allows them to prove and stage the DD^1^. The practical contribution of the Doppler tests enables a positive diagnosis to be still made at the latent phase of evolution when the DD does not have clinical manifestations^6^. The determined echocardiography parameters are shown in Table 2.

**Table 2.** Main analysed echocardiography parameters and the standard values.

Echographical parameter		Mentions related to the mode of determination	Normal values/unit of measurement/expression	
LA volume			≤20 ml	
LV volume		Telesystolic	12–35 ml/m^2^	
		Telediastolic	35–75–90 ml/m^2^	
LV diameter		Telesystolic	24–40 mm	
		Telediastolic	35–75 mm	
LA volume index			30–35 ml/m^2^	
LV volume index			76 ml/m^2^	
LA diameter			28–44 mm	
MAPSE		Indicator of LV longitudinal curtailment extent	normal ≥12 mm	
TASPE		Systolic “excursion” of the tricuspid annular plane	≥20 mm	
Relatively thickness of	SIV	Formula: 2DDSIV/DDLV	Women: 6–11 mmMen: 6–12 mm	media: 9 mm
	LVPP	Formula: 2DDPPS/DDPPS		
LV mass		Formula Devereux R.B., modified	175 ± 30 gram	
LV mass index		LV mass to body surface ratio	Women 75–97 g/m^2^	
			Men: 115 g/m^2^	
Transmural flux	E wave speed	Maximum velocity of mitral flux in protodiastole	50–100 cm/sec	
	A wave speed	Maximum velocity of tardive diastolic filling	20–60 cm/s	
	E/A ratio	Normal LV diastolic function (normal mitral flow)	1–2	
		Delayed relaxation mitral flux	≤0.8	
E′ speed	Lateral ring	Normal LV diastolic function (normal mitral flow)	>10 cm/s	
		Delayed relaxation mitral flux		
	Septum ring	Normal LV diastolic function (normal mitral flow)	>15 cm/s	
		Delayed relaxation mitral flux	<7 cm/s	
E/E′ ratio		Permits the identification of the pressure in pulmonary capillaries	≤8; (ideal >10)	
TRIV (LV isovolumetric relaxation time)		Normal LV diastolic function (normal mitral flow)	70–90 ms	
		Delayed relaxation mitral flux	>90 ms	
TDE (E wave deceleration time)		Normal LV diastolic function (normal mitral flow)	160–240 ms	
		Delayed relaxation mitral flux	>240 ms	

LA: left atrial; MAPSE: mitral annular plane systolic excursion; SIV: interventricular septum; LVPP: LV posterior wall; DDSIV: diastolic diameter of the interventricular septum; DDPPLV: LV posterior wall in diastole; DDLV: LV diastolic diameter.

Formula Devereux R.B. used to calculate the mass of LV^6^:

$$0.8\left[1,04{\left(\text{DDSIV}+\text{DDPPVS}\right)}^{3}-{\;\text{DDVS}}^{3}\right]+0.3.$$

In order to include in the research sample only those patients with chronic ischaemic cardiopathy and preserved left ventricular ejection fraction (LVEF) (≥50%) (PRESYF), it was necessary to evaluate the global systolic function of LV. For this purpose the Simpson modified method was applied, with the use of the following formula:

LVEF=(VTDLV−VTSLV)×100/VTDLV.

On the next stage of positive diagnosis establishment, for the selection of the required study sample, in the conditions of confirmed normal ejection fraction values and absence of any associated diseases, excluding of course the ischaemic cardiopathy, the LV diastolic function was assessed, practically the existence of DD. The DD diagnosis was made on the base of the following 3 obligatory criteria given by the European Study Group on Diastolic Heart Failure:

1. Signs and symptoms of CI:
    • Symptoms: dyspnoea and/or arterial hypertension during effort states.
    • Signs: gallop rhythm, respiratory sounds – pulmonary crepitation and even acute pulmonary oedema.
2. Normal or slightly affected systolic function.
3. Abnormalities in diastolic relaxation, filling, distensibility, or stiffness^17^ ^,^ ^18^.

The successive executions of the aforementioned phases enabled the selection of sample patients with a positive diagnosis of DD with PRESYF, with the unique aetiology of chronic ischaemic painful cardiopathy (stable angina pectoris of effort).

**Figure 1.**
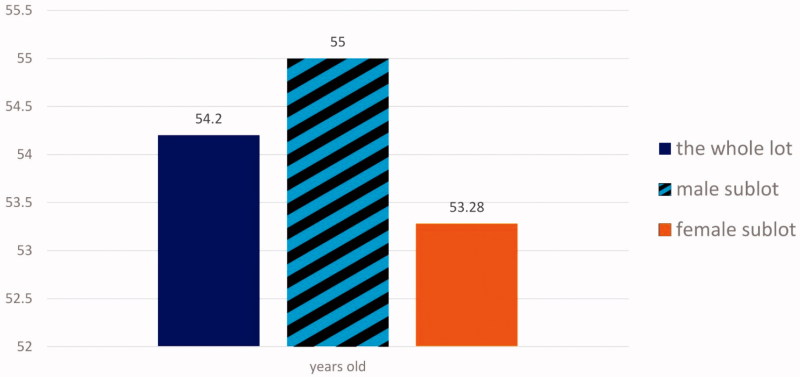
Average age of the patients from the research sample; comparison between the subsamples depending on sex.

An essential criterion imposed for including patients in the study sample was their age. It was necessary because the medical literature unanimously acknowledges the interdependence between CI by DD and age, acquiring variable rates of this positive age-correlation (in general 55–65%). The research did not cover people older than 60 years, hence, the average age of the sample was 54.2 years old with subsamples according to Figure 1.

The lack of voluntary participation of patients older than 60 years is based on the scientific demonstrated argument that although elderly people experience precocious alterations in the ventricular passive filling, the global filling is conserved due to the arterial systole contribution. It is universally accepted that after the age of 60, the participation share of the atrial systole in the ventricular diastolic filling reaches 30% of the cardiac debit^2^ ^,^ ^3^. In addition, the elderly suffer modification in the values of some of the analysed echographical parameters, such as the E/A ratio or the E′ velocity^6^.

The body mass index (BMI) of the patients accepted in the current research presented variations within the margins of 26 ± 3 kg/m^2^, which corresponds to a body mass value considered normal or with 10% ponderal excess and associated with a minimum risk^3^. The introduction of this criterion was obligatory to avoid the possibility to influence some of the analysed echographic parameters.

In addition, for the purpose of the study, there were not accepted patients with arterial hypertension or with tachycardia/tachyarrhythmia, because these diseases, as well as the age, are predictors for some of the examined echocardiographic parameters – E/A ratio and TDE^19^.

## Results

The patients from the study sample underwent laboratory tests on venous blood, the outcome of which is shown in Table 3, among the sample distribution of the determined values.

**Table 3.** Serum tests results of the patients analysed in the present research.

Parameter		Reference values		Amount of patients	
		Unit	Domain (normal/pathologic)	Abs. val.	%
Hb	Men	g/dL	Normal: 14–17.5	56	68.5
	Women		Normal: 12–16	35	31.5
Ht	Men	% mL	Normal: 40–48	56	68.5
	Women		Normal: 36–42	35	31.5
ESR (1h)		mm	Normal: <20	91	100
Blood glucose		mg/dL	Normal: 73–110	91	100
Cholesterol	Total	mg/dL	Normal: <200	11	12.1
		mg/dL	Borderline: 200–240	16	17.6
		mg/dL	High risk: ≥240	64	70.3
	HDL	mg/dL	≥40	11	12.1
	Total cholesterol/HDL cholesterol	Low risk: 3.3–4.4		12	13.2
		Low risk: 4.4–7.1		31	34.0
		Moderate risk: 7.1–11		38	41.7
		High risk: ≥11.1		9	9.8
	LDL	mg/dL	Optimal: <100	7	7.7
		mg/dL	Optimal to limit: <129	10	10.9
		mg/dL	Borderline:130–159	11	12.1
		mg/dL	High: 160–189	29	31.9
		mg/dL	Very high: ≥190	34	37.3
Triglycerides		mg/dL	Normal: <150	10	10.9
		mg/dL	Borderline: 150–199	18	19.8
		mg/dL	High: 200–499	49	53.8
		mg/dL	Very high: ≥500	14	15.4
High-sensibility C reactive protein (hs CRP)		mg/L	Low risk: <1	17	18.3
		mg/L	Medium risk: 1–2.9	46	50.6
		mg/L	High risk: ≥3	28	30.8
Myeloperoxidase		U/mL	Negative level: <7	24	24.1
		U/mL	Intermediate level: 7–10	29	31.6
		U/mL	Positive level >10 U	38	41.5
Liver alkaline iso-phosphatase (ALP)		U/L	Normal: 30–120	91	100
Glutamic-oxaloacetic transaminase (GOT or AST)		U.I./L	Normal: 5–40	91	100
Glutamate-pyruvate transaminase (GPT or ALT)		U.I/L	Normal: 7–56	91	100
Creatinine		mg/dL	Normal: <1.2	91	100

Whereas the main study objective was the MPO serum levels research, it was obligatory to include its determination among the conducted laboratory investigations. The reasoning of its establishment was that increased serum concentrations of this enzyme in patients with DD are an indicator for the existence of an inflammatory process that develops not only on the coronary level but also on myocardial one in the hypoxic/ ischaemic state.

The analysis approach applied for quantifying MPO was the immunoenzymatic fluorescence method (FEIA) that uses as an antigen purified MPO enzyme acquired from human neutrophils. The method allows the anti-MPO antibodies detection, which according to the specialised literature data are implicated particularly in the vascular inflammatory pathogenesis, because of their role as co-participants responsible for the set of vascular dysfunction by atherosclerosis.

The data from the analysed parameters that will be further referred to in Discussion section were evaluated using *t*-Student’s test, with statistical signification being affirmed for values of *p* < .05.

## Discussion

From the symptomatological point of view, the diagnosis of DD among the patients in the research sample was based on the following (Table 4):

**Table 4.** Symptoms incidence and defining signs of DD in the study sample.

Symptom presented	Incidence expressed in	
	Absolute amount of cases	%
No symptoms*	21	23
Dyspnoea at effort	29	31.8
Arterial hypotension at effort	17	18.6
Dyspnoea + hypotension (both at effort)	24	27.1

*Patients had stable angina pectoris, but none of the symptoms that define DD.

For the patients with chronic ischaemic cardiopathy but with no evident symptomatology (asymptomatological patients), the DD diagnosis was established based on the data from the echo-Doppler test.

To establish the diagnosis of DD according to the three recommendations of the European Study Group on Diastolic Heart Failure and to highlight abnormalities in relaxation, filling, distensibility, or stiffness of the LV the values of the echographical parameters from the list below were interpreted:

- LA volume index
- LV mass index
- Relative thickness SIV, respectively, PPVS
- E-wave speed
- Transmitral flux, calculated by the E/A ratio
- E-wave deceleration time
- TDE
- E/E′ ratio between the E-wave and E′-wave speed^5^ ^,^ ^20^.

With the use of “symptoms and signs of CI” criteria recommended by The European Study Group on Diastolic Heart Failure, the patients from the study sample were sorted in different classes of inotropic deficiency, after the classification of NYHA (Table 5).

**Table 5.** NYHA classification of cardiac insufficiency^10^.

Class	Symptoms defining the class
**I**	No symptoms at effort
**II**	No symptoms at resting state.Slightly limited physical activity; symptoms appear at greater levels of physical activity than usual (daily).
**III**	Usual (low) physical activity induces symptoms.Symptoms disappear at resting state.
**IV**	Severe limitation in physical activity, symptoms appear even atresting state.

The basic criterion of the NYHA classification is the symptomatology, which made it quite difficult to sort the participants in the research sample in the respective classes of the myocardial contractile deficit, considering that patients with DD and preserved systolic function present imprecise symptomatology – a particular aspect that is referred to in the specialised literature. In the same respect, it can be mentioned the existence of patients in the study that lacked symptoms of CI.

In accordance with the NYHA classification aspects, the classes of the myocardial contractile deficit were as shown in Table 6 ^1^ ^,^ ^21^ ^,^ ^22^.

**Table 6.** Incidence of myocardial contractile deficit cases in the study, based on NYHA classification criteria.

Class of contractile deficit	Incidence expressed in	
	Absolute amount of cases	%
I	21	23
II	48	52.8
III	22	24.1

As evaluating the signification of MPO serum values is the main objective of the study, it was necessary to establish the blood level of this enzyme. Serum concentrations above the negative result of 7 U/mL were detected in 67 patients (73.1%). The amount of cases is 2.7 times higher than the negative result, which corresponds to a positive correlation in 2 from 3 situations (Table 1).

Even if the number of cases in the current study is considered relatively small, the results in determining MPO serum levels represent a strong argument to support the fact that MPO can be used as a biomarker for diagnosis and risk estimation of DD, occurring as chronic ischaemic cardiopathy with conserved systolic debit volume.

To endorse the previous suggestion there is also the protocol recommended by Cleveland Heart Lab to use the multi-biomarker CVD Inflammatory profile test with the purpose of DD diagnosis. The latter includes two of the serum parameters measured in the present study, namely (MPO and hs-CRP), but also 4 additional ones: oxidised lipoproteins, A_2_ phospholipase (lp-PLA_2_), F_2_-isoprotan, and microalbuminuria^23^.

In comparison with the standard values the following echographic parameters showed pathological deviations in the patients of the research sample:

- E-wave deceleration time, TDE, increased in 83 cases = 91.1%.
- Relative thickness SIV and PPVS, respectively, in 35 cases = 71.4%.
- LV mass – increased in 56 cases = 61.5%.
- LV index mass – increased in 59 cases = 64.8%.
- E/A ratio – abnormal in 78 cases = 85.7%.

The next stage of the study consisted of identifying possible correlations between MPO serum concentration and the determined values of some of these echocardiography parameters. Therefore, the serum MPO was compared with TDE, LV mass index, and E/A ratio. Therefore, the direction of level variation of the circulating MPO serum concentration variations was compared with that of the following echographic parameters’ variations: TDE, LV mass index, and E/A ratio.

Referring to DD, the medical literature reports that the first echographic parameter to become abnormal by increasing its value is TDE. Incidence of values above 240 ms reaches 91.1% in the study sample, occupying the first place among the selected echographical parameters. The correlation between the TDE values and the MPO serum concentration showed a ratio of 83 cases/67 cases = 91.15/73.1%.

The concordance in the proportion of ¾ of the analysed cases between these two parameters allows elaborating the following statements:

- Patients with DD and PRESYF MPO serum concentration above 7 U/mL is significantly correlated statistically with relaxation and even ventricular compliance modifications in the advanced stages of the disease. The thoroughgoing study based on the results from the present research, by conducting investigations on numerically significant samples of patients, possess high chances of proving and accepting the utility of serum MPO as a biomarker for DD with PRESYF.
- The fact that in 16 cases were reported prolonged TDE values while presenting normal MPO serum concentration (<7 U/mL), can be interpreted as caused by:
- the increased sensibility of the TDE parameter in identifying the existence of delayed relaxation in comparison with the circulating MPO levels that raise at a later phase during the disease evolution.

The large accordance rate between these two parameters can also be used as an argument for supporting their association, as a complex biomarker, the information rendered by the two in medical practice being useful for the fact that they support each other, at the same time completing each other.

In medical practice, the elevated SIV, PPVS, LV mass, and LV mass index values are considered an illustration of concentric cavity remodelling, but at the same time also an indication for myocardial hypertrophy, as a functional expression of modifications in the LV myocardial compliance^10^ ^,^ ^24^ ^,^ ^25^.

In the current research, the increased incidence of LV mass index (59 cases = 64.8%) was corroborated with that of LV walls relative thickness (SIV + PPVS) reported in 65 cases (71.4%), which had moderately higher values than those accepted as normal. Without taking into account the patients’ sex, the echographic existence of modifications in the aforementioned variables indicates that in at least 2/3 of the DD cases with PRESYF the ventricular concentric hypertrophy was present.

In all 59 cases with increased LV mass index, there was also detected an increase in the serum MPO concentration ≥7 U/mL.

The 100% accordance between the two parameters makes it possible to consider that the circulating MPO level can be recognised as an indicator for the onset of LV myocardial hypertrophy developing process and therefore can be accepted as a marker for DD.

## Conclusions

Despite DD being a relatively light medical condition, it possesses a prognostic importance, proving the existence of certain cardiovascular risk, with the possibility of installing a clinical syndrome of CI. Therefore, the interest in developing new laboratory and paraclinical non-invasive examinations for the detection of DD as early as possible is completely justified.

The current study shares this aim and the obtained results allow the statement that MPO serum level, independently or in correlation with the echographic data, can become a useful investigation for the medical practice as a biomarker of DD with PRESYF.

During the clinical track of DD with PRESYF evolution, between the two respective parameters, the informational value of the enzymatic serum concentration has a relatively delayed sensibility, whereas there are echographically evident signs of delayed or incomplete relaxation. Consequently, if it is not possible to perform the echocardiography, MPO serum concentration dosage is useful in diagnosing and evaluating the risk for DD with PRESYF.
